# A comparison of recall methods for high-stress critical incidents in police training

**DOI:** 10.1016/j.heliyon.2024.e36562

**Published:** 2024-08-19

**Authors:** Michael John Roscoe, Suzanne Gough, Robin Orr, Oliver Baumann

**Affiliations:** a Australian Federal Police, Canberra, ACT, 2600, Australia; b School of Psychology and Counseling, Queensland University of Technology, Musk Avenue, Kelvin Grove, Queensland, Australia; c Tactical Research Unit, Bond University, Robina, QLD, 4226, Australia; d Faculty of Health Sciences and Medicine, Bond University, Robina, QLD, 4226, Australia; e Faculty of Society and Design, Bond University, Robina, QLD, 4226, Australia

**Keywords:** Memory, Police, Central, Peripheral, Interview, Timing

## Abstract

Police officers often face critical incidents involving armed offenders, requiring the use of force to ensure safety. Eyewitness accounts, including those from officers, are crucial in the justice system but can be unreliable. Techniques such as self-authored statements and structured interviews are used to gather information, but their efficacy in high-stress situations is unclear. Previous research suggests that heightened arousal during memory encoding enhances recall, particularly for central details. This study compares recall methods (statements vs. interviews) for police officers in high-stress versus no-stress situations, focusing on central and peripheral event details. Officers participated in a simulated high-stress incident, providing memory data through both methods. Overall, no significant difference was found in memory scores between the techniques. However, analysis revealed significant differences favoring structured interviews for peripheral information. Recall that central information remained consistent across methods. These findings highlight the need for careful methodology when examining memories formed in stressful contexts.

## 1 Introduction

Police officers are responsible for dealing with critical, potentially life-threatening, incidents that they must accurately recall for subsequent legal proceedings. Critical incidents include, but are not limited to, events where police officers confront armed offenders. These events may involve taking a life to protect their own or save another. Critical incidents are sudden, novel, intense, and relatively short events that require an immediately effective response, disrupt goal-based behavior and are psychologically stressful. They are prototypical "emergency" situations where the consequences of poor performance are immediate [1].

To obtain information from eyewitnesses, police officers use a range of techniques. First, they are often taught and encouraged to use their notebooks to construct a self-authored statement in the early stages after a critical incident. Another widely used technique is a structured interview, which is a type of structured conversation that encourages detailed reporting, including contextual information. However, this technique has not been specifically crafted for active responders of stressful critical incidents in Australia. The role of eyewitnesses, including police officers, is central in the criminal justice system. However, their accounts can be inaccurate and lead to wrongful convictions [2]. Therefore, it is essential to establish the comparative reliability of different recall and recording practices in assisting witnesses in accurately reconstructing events, particularly when the event occurred under conditions of high stress. In light of the modern use of body worn cameras (BWC) the video footage they collect may act as an "aide memoire" similar to how notes are made. However, the arbitrary viewing by officers of BWC footage before official investigation can be problematic when considering the constructive nature of memory recall and the occurrence of perceptual distortions prevalent in high stress environments against the objective reality of video footage [3].

Studies have shown that elevated arousal during encoding increases the chance of remembering a particular stimulus [[4], [5], [6]]. Most interestingly, some studies have demonstrated that arousal differentially affects the encoding of central and peripheral aspects of events and scenes [[7], [8], [9], [10], [11], [12]]. For instance, Wiemers and colleagues [13] employed the Trier Social Stress Test (TSST) to induce heightened arousal. The researchers found that participants remembered central visual details of the stressful episode, such as interactions with the test examiners, better than central visual details of the nonstressful situation. Additionally, Wiemers et al. [13] found that stressed participants had a more accurate memory for central visual details than nonstressed participants, including remembering the faces of the test examiners better than the no stress controls.

Previous studies that have contributed to understanding memory recall for stressful/distressing events have tended to examine memory after exposure to gruesome photographs of accidents or surgeries or by using emotional words [10,[14], [15], [16], [17]]. While these studies show changes in recall ability when comparing neutral to simulated stressful scenes, they conflict with the concept of arousal congruent performance (ACP) [18]. According to ACP, the stress induced must be representative of the environment or context in which the memories are formed. In a police officer's case, the stress is directly caused by the to-be-remembered information, such as a firearm [19]. ACP proposes that there should be a meaningful connection between the arousal state induced and the performed task-relevant actions that are evaluated, a condition termed by Hanoch and Vitouch [18] as ecological rationality.

Previous studies have investigated the effects of various information gathering techniques in the eyewitness context and the impact of stressful situations on the memory of police officers [[20], [21], [22], [23]]. However, our present study adopts a unique approach. It is the first to integrate both aspects within a single investigation, specifically comparing two common types of information gathering among police officers under realistic stress scenarios, while also exploring the differential effects of stress on retention of peripheral and central information.

The current study aimed to investigate the comparative effectiveness of the two most commonly used methods of recall (i.e., statement vs. interview) of police officers' memories immediately after an ecologically valid simulated high-stress vs. no-stress situation. Importantly, the effectiveness of the recall method will be compared separately for central and peripheral information of the critical event.

## 2 Method

### 2.1 Participants

Participants were members of an Australian police service (*n* = 42; mean age = 35 ± 4.3 (range = 28–44) years). All participants except one were male. Each participant had a minimum of two years of policing experience (mean years' experience = 8.5 ± 1.23 (range = 5.5–15) years), and all provided written informed consent. The study received approval from the ethics committee of Queensland University of Technology (1,000,000,360). G-Power analysis suggested that a sample size of 52 participants would be needed to reach 80 % power for comparing two independent groups. Aiming for a large effect size in a study of memory processes in police officers is justified because larger effects are more likely to have practical significance, be robust and replicable, and facilitate changes in procedure. Although the sample size did not reach the optimal level (due to the small number of trained officers and operational commitments), it still provided reasonable statistical power for an applied study, and the results should be interpreted in light of this limitation. This limitation in cohort sizes has been noted in previous studies involving specialist police personnel [22,24].

### 2.2 Experimental setting

**Fig. 1:**
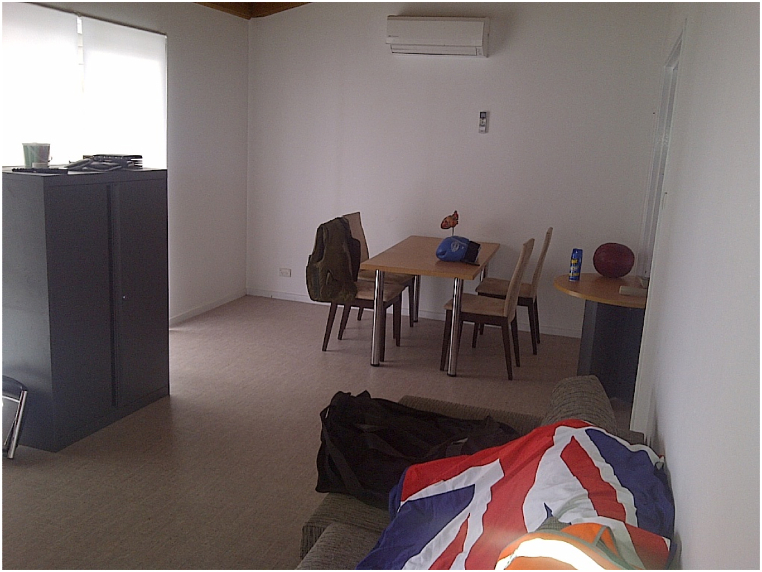
Overview of the experimental room inside the police training complex resembling a domestic premise.

**Fig. 2:**
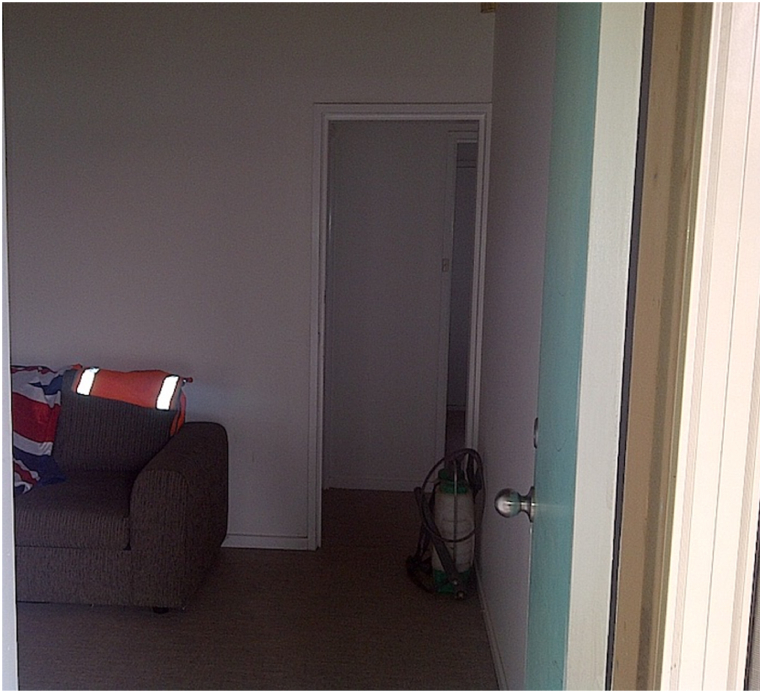
View from the main front door providing entry into the experimental room.

The study utilized a specially prepared room in the police training complex (see Fig. 1, Fig. 2). *Environment:* Scenario training room designed to replicate domestic premises. The same experimental room was used to construct two experimental conditions: stress and no stress.

*Props:* The room contained several miscellaneous items, including a flag draped over a couch, a weed sprayer, a barbell, a boxing glove, a medicine ball, and a telephone with the receiver resting on the table. These items were randomly placed in the room to clutter the visual field. The number of items situated in each condition (stress/no-stress) to be recalled by the participant was equal. In the stress condition, weapons and threatening movements were visible, while in the no-stress condition, simulated participants held a magazine and envelopes.

*Video recording equipment*: Two fixed cameras captured a general panoramic view of the room from the direction of the simulated participants and the participants.

**Fig. 3:**
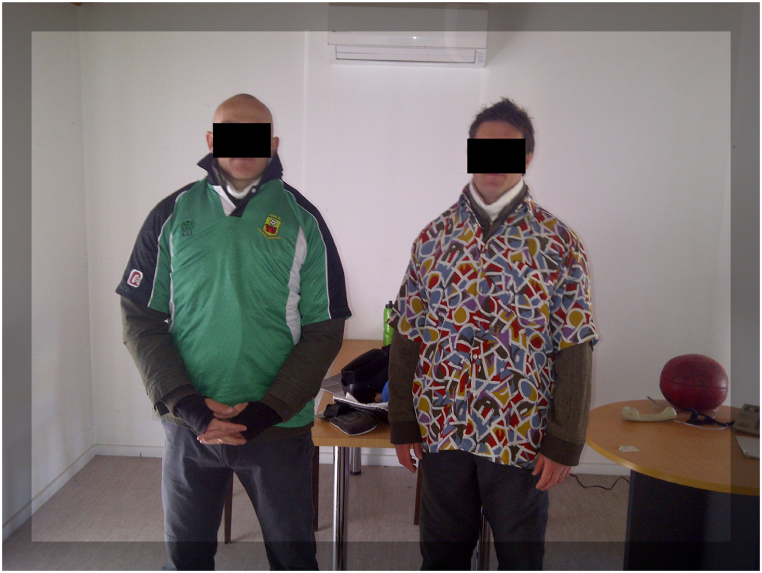
Simulated participant garments.

**Fig. 4:**
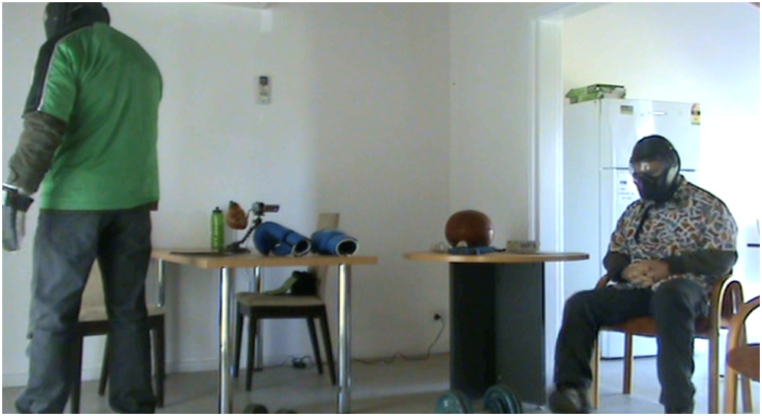
Position of simulated participants prior to confronting the police officer participants.

**Fig. 5:**
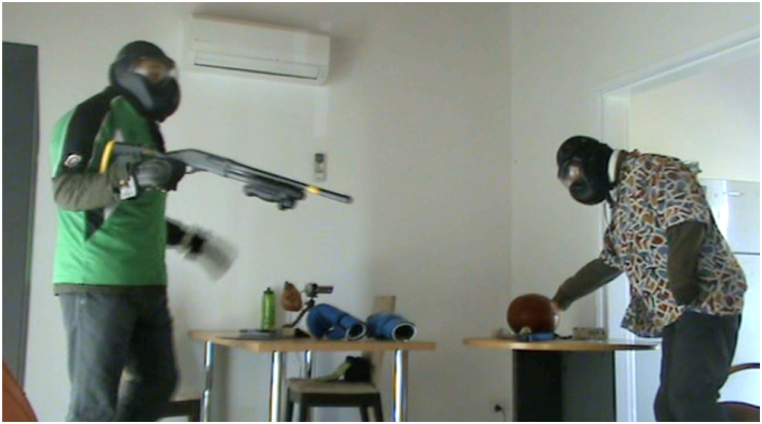
Position of simulated participants displaying threat toward police officer participants.

*Stress condition (*Fig. 4, Fig. 5*):* Two simulated participants wore distinctive clothing (Fig. 3). A simulated participant is a person or actor trained to portray a specific role within the scenario. Protective equipment was only used in the stress condition consisting of under garments of simmunition body armour and protective masks and gloves. Their faces were not clearly visible. Two weapons were used, a pistol and a shotgun, with a single weapon available for each simulated participant. Upon entering the room, the participants saw one simulated participant standing in the left corner of the room facing the wall and holding a shotgun in their right hand that was initially occluded by their body (Fig. 4). The second simulated participant was sitting on a chair in the opposite corner facing toward a table with the pistol clearly visible on the table but just outside of the arm reach.

**Fig. 6:**
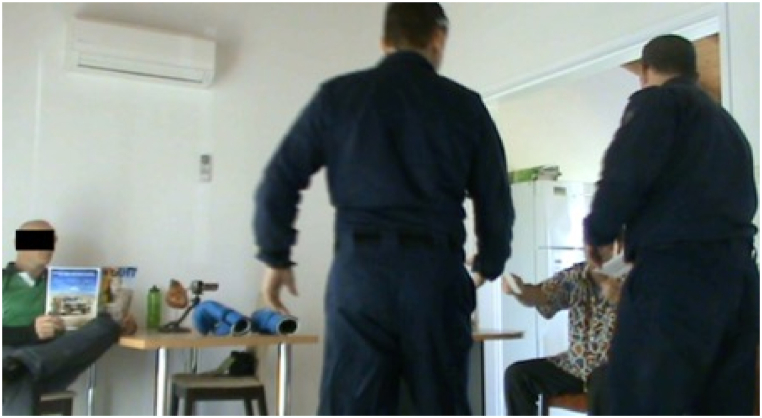
Participants in the no stress condition receiving envelopes.

*No stress condition* (Fig. 6*):* In contrast, the two simulated participants wore identical clothing but without protective face helmets and under armour vests (Fig. 6). The occupational health and safety rules of police force-on-force training stipulate that full protective equipment be employed in a simulation training environment. Therefore, if a participant identifies simulated participants without protective equipment, it signals a ‘no threat’ scenario. In this specific scenario, both simulated participants were sitting on chairs near a table in the same vicinities as in the stress condition (Fig. 6). In the no-stress condition, the simulated participants' hands were visible, either by holding a magazine or holding the envelopes, ensuring that participants did not see anything threatening that would produce negative arousal. Simulated participants in this condition did not wear masks so that facial features were visible and capable of being recalled.

*Participants uniform: (protected information).* Participants were required to wear blue overalls with a utility belt holding the officer's operational equipment, including a Simmunition prepared firearm, handcuffs, baton, spare Simmunition rounds in magazines, OC gas, and a Taser. In addition, personal protective equipment (PPE) in the form of neck guards, groin protection, gloves, helmets, and facemasks were worn. Specialized training ammunition known as FX Simmunition marking ammunition (GD-OTS Canada Inc., Repentigny, QC, Canada) was used to provide realism by providing a round that allows engagement of simulated participants (and officers) with no injury if they are suitably protected. Simmunition is a nonlethal projectile contained within a thin plastic sabot that carries a soap/paint-type substance. For those in the stress group, all exposed skin was covered to protect the participant from the effects of Simmunition projectiles.

### 2.3 Design

The independent variables of the current study included the stress level experienced by police officers and the recall method. Specifically, participants were exposed to ecologically valid simulated high-stress and no-stress situations. The stress level was manipulated by either exposing participants to scenarios involving threatening situations with weapons and simulated threatening movements (high-stress condition) or neutral scenarios with nonthreatening gestures and objects (no-stress condition). The recall method was manipulated by either having participants immediately provide a self-authored statement followed by a structured interview after a delay or participate in a structured interview immediately and then prepare a self-authored statement after a delay. While this study does not investigate timing effects, it was taken from a larger study testing the time factor between 0 and 48 h. We describe the timing factor to give the reader a comprehensive overview of the design. The dependent variables focused on the effectiveness of the recall method in retrieving central and peripheral information related to the critical event. Memory recall was assessed immediately following the scenarios using structured interviews or statements, and central and peripheral memory scores were analyzed separately. The design aimed to compare the recall methods under different stress conditions and assess their efficacy in retrieving both central and peripheral details of the event.

### 2.4 Scenario procedure

Participants were randomly assigned to one of two groups: a stress group and a no-stress group by the author. Within each group, participants were then randomly paired. Following randomization, an event briefing was provided, which was tailored to the respective condition (stress/no-stress). Participants in the stress condition were instructed to wear protective equipment, while those in the no-stress condition were instructed to wear uniforms without protection. The scenario conditions and objectives varied as per Box 1. The scenario durations were identical, between six and 8 min for each pair of participants.

Box 1 **Scenario conditions and objectives.**

**Table:** 

**Stress Condition scenario objective(s): Memory subsequent to a use-of-force threat situation.** The participants in the stress condition were required to conduct clearance drills of rooms in nearby buildings in the training complex prior to reaching the building containing the experimental room. They were unaware of the location of the simulated participant offenders and unaware that one of the rooms was part of a study. The room clearance drills are normal training for police. In the experimental room, a simulated participant was situated holding a shotgun in their right hand, facing the left corner of the room (only visible after entering through the front entrance door) (Fig. 4). As the main door opened, this simulated participant turned to confront the participants, pointing the shotgun toward them. A second simulated participant, initially sitting on a chair on the right side of the room, then stood up and moved their hand toward a pistol placed on a table (Fig. 5). The timing and movements of the simulated participants were determined by the opening of the front door. The police participants responded to the threats and continued their actions until the scenario was considered safe (e.g., move to handcuffing). At this point a call of "End Ex" given by the police instructors stopped the scenario. **No-Stress Condition scenario objective(s): Memory subsequent to a neutral situation.** In the no-stress condition, the same simulated participants as in the stress condition moved and occupied the same areas of the room but with nonthreatening gestures and objects and without protection equipment. On the left side of the room, a simulated participant was sitting next to the table holding a magazine folded to reveal the front-page information. A second simulated participant seated on a chair on the right side of the room greeted the participants before handing them sealed letters, which they were asked to open outside of the room. The participants then left the room and carried out the instructions to read the contents of the envelope (informed consent forms) and were then approached by the author to take part in the study.

Alt-text: Box 1

**Data collection:** After the scenario, as the participants left the building containing the experimental room, they were asked to take part in the research. Sealed envelopes containing the informed consent forms were available for each pair of participants. Those from the stress condition were handed informed consent forms to read and sign outside the experimental room. Those in the no-stress condition had received consent forms in sealed envelopes as part of the scenario. Written and verbal consent was obtained from all participants. Verbal consent extended to participation in research-specific interview questions that replicated normal procedures when being interviewed by police internal investigations.

The participant information indicated that they were involved in research into visual search techniques. This minor deception was necessary to ensure that participants' postevent activities would not affect their memory process of prior events before a further interview was designed to take place 48 h later. Participants were not aware that they would be reinvestigated 48 h later. This procedure is similar to a normal police investigation, as investigators gain further information and reinterview witnesses at a future stage. In both conditions, participants were fully debriefed on the recent activity and nature of the study and verbally asked to give informed consent in addition to signing the written request.

A coin toss determined whether a person from each pair completed an interview or a written statement, with the other person assigned the other method of recall. Therefore, in each pair, one person completed an interview and the other a statement immediately following the scenario. Prior to the interview, participants were randomly assigned to either List 1 or List 2 of the structured interview by a coin toss.

In both the Stress and No Stress conditions, participants were provided with time for reflection before specific questions were asked, following a "free recall" process, during which participants recounted the event from start to finish. The structured interview is designed to withstand the effect of stress by extracting information specifically related to memory processing under stress. In the stress condition, the participants received their informed consent forms upon leaving the experimental room, which was the final room cleared and the end of the exercise. A laptop computer with video recording software was used to record the interviews with the participants.

**Structured Interview:** The structured interview included a free recall component followed by 20 structured questions. Two unique lists of these questions were created, and they were fully randomized in the order of presentation *(see Supplementary File A)*. The questions aimed to distinguish information relating to the central and peripheral aspects of the event but did not include leading questions or information revealing how the scenario was resolved. Questions were direct and designed to obtain positive confirmation as well as an explanation for a certain behavior (e.g., "Did you use force? And if so, why was that necessary?"). The questions were open-ended to obtain details of both central and peripheral information.

**Self-Written Statement:** Those participants selected to complete a written statement entered a room and completed their statement on a blank sheet of paper without any prompting or questions in the same manner they would conduct a handwritten statement in a real-world policing context.

**Grading:** Memory recall was coded using a grading process that involved a reduced set of Nideffer's quadrants of attention [25]. Recall was graded for its appropriate fit into one of Nideffer's quadrants of attention using only the categories of external narrow (central information) or external broad (peripheral information) (see Supplementary File C). External narrow information was defined as "information tied to the main character or theme of the event," and external broad information was defined as "information unrelated to the main character or theme of an event" [25]. The graders were also assisted by the explanation that central and peripheral information refer to the difference between a focus that is associated with the source of the arousal (i.e., the gist of the event and its central details) and a focus that is spatially peripheral or irrelevant to the source of the arousal (peripheral details). For example, if the participant recalled the weapon, this observation was classified as an external narrow focus (central). Similarly, if the participant recalled aspects of the room, this was graded as an external broad focus (peripheral). Nideffer's use of the word "focus" was regarded in the first instance as a reflection of the officer's attention, but when transformed into a memory and recalled, it becomes a source of information and categorized as either central or peripheral information and used in this fashion in the studies.

Graders completed a specially designed grading chart for each participant's recall at immediate recall time. Each of the transcripts of the interviews (prepared by the lead author) and statements were checked for accuracy by comparison against video taken of each pair of participants. Memory recall was scored as either central or peripheral information and as either correct or not correct using the grading chart *(see Supplementary File B)*. The chart described in detail the objects, persons, environment, actions, sounds, and weapons (stress condition only) in the experimental room. The domains were defined in the following manner: (i) Object: any mention of an artifact not part of a person's description and not a weapon; (ii) Person: descriptive detail of the person's features, including clothing; (iii) Environment: any reference to the lighting or climatic condition, including an explanation of its effects on the officers' efficiency; (iv) Action: physical movements, actions of the role-playing offenders, including partner officer, excluding verbs related to sensory perception; (v) Sounds: any sound heard, including dialogue of any nature; and (vi) Weapons: description of weapons including their orientation (e.g., barrel pointing toward officers). The accuracy judgments were executed by expert law enforcement graders, following the standards typical in eyewitness assessments. For instance, in detailing clothing, a brief description regarding its type and colour sufficed.

Supplementary File C offers an illustrative grading form completed by an independent Grader, empowering them to discern whether objects, actions, etc., align with the classification of central or peripheral as defined by Nideffer. Furthermore, the Grader evaluates the accuracy of these classifications. Notably, the scoring system is based solely on a raw number memory score derived from the summation of individual grading sheet scores, without any proportional adjustments.

### 2.5 Analysis

The design of the study reflected a typical police investigation procedure following a critical incident, where an officer prepares self-authored notes followed by a structured interview after a delay. Alternatively, an officer participates in a structured interview immediately and after a delay prepares a self-authored statement. Recall was tested using interviews or statements.

Descriptive statistics and tests of normality using Kolmogorov‒Smirnov and Shapiro‒Wilk procedures were conducted on central, peripheral, and total memory scores. Histograms and Q‒Q plots were reviewed visually to check for deviations from normal distributions. Levene's test of equality of variances was also performed. Following this, a series of t tests and nonparametric tests utilizing the Mann‒Whitney *U* test were conducted on central, peripheral, and total memory scores.

A manipulation check was conducted to ensure that participants were stressed in the stress condition. This was established by two questions from the structured interview: "How did you feel?" and "Were you stressed or unable to cope?" *(See Supplementary File B).*

Two graders examined the transcripts of the interviews and statement forms. Both graders scored 100 % of the data. Reliability tests were conducted on peripheral and central information taken from the immediate and delayed results. The scale had a high level of internal consistency (Cronbach's alpha of 0.916) based on the grading by Fleiss [26]. Alpha levels for all analyses were set at p < .05 a priori.

## 3 Results

Levene's test of equality of variances indicated that central and peripheral data were significantly different (*p* > .05), and therefore, the sampling distribution was nonnormal. Additionally, visual examination of the histograms and Q‒Q plots of central and peripheral data also confirmed that the data were not normally distributed. Therefore, a nonparametric analysis was conducted for the central and peripheral data.

### 3.1 Total memory scores

The prediction that participants in the stress condition would recall more than participants in the no stress condition was not confirmed (see Table 1); a difference of −1.86 (*BCa* [−4.00, 0.279]) was not statistically significant, *t =* −1.54, *p* = .132, *d =* 0.46).

**Table 1:** Mean scores and standard error of the mean of central (C) and peripheral (P) memory recall at immediate (0) hours, shown separately by the arousal and recall method (interview vs. statement), including overall mean totals.

	Time of Recall				
	Immediate (0-h)				
	Interview *(n = 19)*		Statement *(n = 23)*		Total Overall Means
	C	P	C	P	
Stress (*n* = 21)	18.11(1.76)	6.06 (1.59)	17.38 (1.52)	3.38 (1.38)	11.23 (1.21)
No Stress (*n* = 21)	9.30 (1.67)	11.30 (1.56)	7.55 (1.59)	9.05 (1.44)	9.30 (1.20)

*Note.* Standard Deviation (SD) of the Mean in Brackets. C - Central P - Peripheral.

### 3.2 Stress vs No stress

Two pairs of comparisons were conducted using a nonparametric independent samples Mann‒Whitney *U* test. Peripheral information across the arousal factor differed significantly between the stress (*Mdn* = 4.50) and no stress groups (*Mdn =* 10.00), *U* = 82.00, *z* = −3.50, *p* < .001, *r* = −0.76, a large effect size, CI[2.00; 7.50]. Central information across the arousal factor differed significantly between the stress group (*Mdn* = 18.00) and no stress group (*Mdn* = 8.50), *U* = 386.00, *z* = 4.17, *p* < .001, *r* = 0.91, a large effect size, CI[-13.50;-6.50]. These findings indicated that stress produced greater central recall of the event with less peripheral information, whereas the opposite effect occurred in the no stress control condition.

### 3.3 Method of recall

A Mann‒Whitney *U* test was conducted on central and peripheral memory scores using the method of recall as the grouping variable (Interview vs Statement). This analysis produced a significant result on peripheral recall, *U* = 137.50, *z* = 2.05, *p* = .040, *r* = 0.31, a small effect. A Hodges Lehman confidence interval indicated [−5.00; 0.00] as the difference between medians. Central information recall between Interview and Statement was not significant, *U* = 240.50, *z* = 0.56, *p* = .578, *r* = 0.09 and showed that central information was unaffected by the method of recall. Given these findings, further analysis of peripheral recall was warranted by focusing on the method of recall in the stress and no stress conditions.

### 3.4 Statement vs structured interview in the stress vs No stress conditions

Two pairs of comparisons were conducted using a nonparametric Mann‒Whitney *U* test on the previously significant analysis. Peripheral recall from the No Stress condition by Statement was not significantly different from Peripheral recall in the No Stress Interview condition (*Mdn = 7.00* vs *Mdn =* 10.75), *U =* 66.50, *z =* 0.81, *p =*.426, *r =* 0.18. However, peripheral recall in the stress condition taken by statement was significantly different from peripheral recall in the stress condition taken by interview (*Mdn = 3.00* vs *Mdn =* 6.00), *U =* 85.00, *z =* 2.21, *p =*.028, *r =* 0.48, a small to medium effect, a Hodges-Lehman median difference confidence interval indicated [−4.50;-0.50]. This finding suggests that peripheral recall taken by a structured interview immediately following a high-stress incident produced better recall than a self-authored statement.

### 3.5 Recall scores and errors across domains

All scenarios in the study were filmed, and therefore, the recall remarks of the participants could be examined against the footage. The role-players were scripted to perform in an exact manner, with no variation unless the officer participant did something out of the ordinary, which did not occur. Table 2 provides a detailed view of the mean memory scores and mean error scores for each domain. The description of behavior in the central domain of action produced the highest scores with few errors. The action domain is particularly relevant as it forewarns of imminent danger in the stress condition. Participants focused on the person description domain and produced good recall scores; however, this domain also resulted in the highest number of errors in both the stress and no stress conditions.

**Table 2:** Mean Number of Items Recalled of Central and Peripheral Information, indicated separately according to domain (Actions, Environ, Objects, Persons, Weapons, Sounds), and separately by Time of Recall, Method of Recall and Condition (Stress vs. No Stress). Errors are the total of central and peripheral means in each domain.

	Time of Recall					
	Total Domain		Immediate (0-h)			
	Errors		Interview		Statement	
			C	P	C	P
Stress	0.33	Actions	4.70	0.0	6.08	0.0
	0.0	Environ	0.0	1.30	0.0	0.3
	0.0	Objects	0.0	2.60	0.0	0.58
	0.45	Persons	3.00	0.0	2.50	0.0
	0.18	Weapons	1.80	0.0	1.50	0.0
	0.08	Sounds	2.00	0.0	1.75	0.10
No Stress	0.20	Actions	2.80	0.0	1.90	0.0
	0.10	Environ	0.0	1.20	0.0	0.30
	0.0	Objects	0.0	5.20	0.20	3.50
	0.90	Persons	6.40	0.0	5.80	0.0
	n/a	Weapons				
	0.0	Sounds	1.10	0.20	0.70	0.10

*Note.* Weapons were not used in the No Stress Condition.

## 4 Discussion

The aim of the study was to investigate the comparative effectiveness of the two most commonly used methods of recall (i.e., statement vs. interview) for police officers' memories immediately after simulated high-stress vs. no-stress situations. Importantly, the effectiveness of the recall method was compared separately for central and peripheral information of the critical event. The design of the study, using special operations police in training, was ecologically sound and established a high standard in the memory literature for producing valid data concerning the memory recall of police and is an important strength in this study. It is important to note that the two investigated recall methods are both ecologically valid in policing. However, they vary significantly: one involves written free recall, while the other entails in-person questioning. Despite these differences, the comparison remains ecologically valid in policing contexts, and our aim was to examine the impact of stress on these key information gathering techniques. Additionally, the stress versus no-stress condition significantly features more central information, potentially influencing the shift towards central rather than peripheral information, alongside attentional narrowing.

First, the results indicated that the stress and no-stress conditions had differential effects on recall for central versus peripheral information. More specifically, the recall of central information benefited from higher arousal, whereas this was not the case for peripheral information. This finding reflects the original work of Easterbrook [7], who stated that high arousal narrows attention, which in turn benefits performance on central information because relevant threat information is given better attention, and nonthreatening or less-threatening cues are dispensed with. Therefore, central details of a high stress incident are accurately remembered, whereas peripheral details are not. Additionally, Guillet and Arndt's [27] findings indicated that the association between central and peripheral information was enhanced when the central information created high arousal, as seen in both recall and recognition. They suggest that arousal triggers binding mechanisms that provide associations between the arousing information and its context, both within the object and between objects. Taken together, the peripheral narrowing effect is also reflective of 'tunnel vision' [28].

Most interestingly, this study showed that while recall performance for central information in the stress condition remained unaffected by the methods of recall, peripheral recall taken by a structured interview produced better recall than a self-authored statement. This finding may reflect a preservative consolidation of peripheral information. Walker [29] discussed the 'Action Decrement' Theory, whereby central information is consolidated at the expense of peripheral information that consolidates at different speeds to central salient information. Walker's [29] Action Decrement Theory was largely ignored in the memory literature until supporting neurobiological evidence was published. Diamond et al. [30] and Schwabe and Wolf [31] claim that an inhibitory trace caused by the return of cortisol on N-methyl-D-aspartate receptors prevents their normal processing, placing a pause on peripheral consolidation until important central details are stabilized. The temporal dynamics of stress suggest that peripheral information may be accessible after a delay period, giving time for the stress hormones to subside and highlighting the significance of Walker's [29] action decrement theory.

Memories of peripheral information when officers' use-of-force responses are investigated are bolstered when corroborated by other police officers. Peripheral information of this nature now takes on a much greater importance when investigating critical incidents. Therefore, as this study suggests, choosing the appropriate method of recall may be crucial to the overall investigation.

The unique nature of the participant group including access to police specialist training facilities means that this study is necessarily smaller than typical memory experiments completed in the laboratory and reflects an unavoidable limitation in this study. Considering this, and while larger studies are always preferred, smaller sample sizes are common is this form of environment given the complexity of the events and this study did produce a larger sample size than similar studies [32,33]. Similarly, the lack of female tactical operators makes it difficult to generalize the findings across gender with these limitations again notes in the wider research [33]. While it would have been preferable to analyze interactions using a MANOVA or mixed model, given the relatively modest sample size, we chose a univariate approach to maintain statistical power.

The design was ambitious given the low sample size, but the intent was to reflect real-world policing situations. An improvement to manipulation checks for future studies should confirm the presence and effects of stress by empirical means such as heart rate measures or blood catecholamines rather than self-report. Overall, the results indicate that stress and the method of recall differentially affect the type of material being recalled. There may not be a 'one size fits all' solution to the problem of using one technique to investigate critical incidents at one time point, as Porter et al. [34] suggested. The implications for investigators of critical incidents are to consider the differing effects of stress on memory and tailor the timing and method of recall according to the essential aims of the investigation.

## 5 Conclusion

Our study indicates that stress and recall methods have distinct impacts on the recall of central and peripheral information among police officers. Higher arousal enhances the recall of central details, while structured interviews yield better recall of peripheral information. The accurate and comprehensive recall of peripheral details by police officers is vital for effective investigation and legal proceedings. Our findings underscore the importance of investigators in critical incidents considering the varying effects of stress on memory and customizing the timing and recall methods to align with the specific objectives of the investigation.

## Data availability statement

Data associated with this study has been deposited at Open Science Framework: https://osf.io/c2mgb/?view_only=a4307d0b42e74f319e24a56ddf67b8e8.

## CRediT authorship contribution statement

**Michael John Roscoe:** Writing – original draft, Methodology, Investigation, Formal analysis, Data curation, Conceptualization. **Suzanne Gough:** Writing – review & editing, Validation, Supervision. **Robin Orr:** Writing – review & editing, Validation, Supervision. **Oliver Baumann:** Writing – review & editing, Supervision, Methodology.

## Declaration of competing interest

The authors declare that they have no known competing financial interests or personal relationships that could have appeared to influence the work reported in this paper.
