# Supplementary material for: A comparison of recall methods for high-stress critical incidents in police training
Source: Heliyon. 2024 Aug 19;10(17):e36562. doi: 10.1016/j.heliyon.2024.e36562 (PMC11387239; doi:10.1016/j.heliyon.2024.e36562)
Supplement: Multimedia component 1 [file mmc1.docx]

**Supplementary File A**

Questions List One

Confer Group – YES/NO

Number Identifier …………………..

This interview is conducted between _________________________________

and _________________________.The time is now _______________.The

date is _________________.

*Do you agree to take part in this interview?*

*Did you confer with your partner? _____________________________________*

***Free Recall Stage***

*Explain the incident from the time that you entered the room.*

***Questions***

CQ9. What were your main thoughts during the activity?

*CQ10.Was your use of force decision successful?

CQ3. Describe any sounds you heard.

PQ17. Can you describe what the room looked like?

PQ14. What was the lighting like in the room?

CQ2. What did you observe?

*CQ8. Did you use force? And if so why was that necessary?

CQ6. Describe any persons you observed.

*PQ12.Where there any shots fired in the incident?

PQ16. Were you feeling stressed or unable to cope at any time?

PQ19. Did you say anything to your partner?

CQ1. When you entered the room, what did you immediately focus on?

CQ4. How did you feel?

*CQ5. Can you describe any weapons you may have seen?

PQ13. What was your partner doing when you were in the room?

PQ18. What items were in the room?

PQ11. How far away where you from the persons?

PQ20. How long did the event take?

PQ15. Describe any other people present.

CQ7. Describe your movements in the room.

CQ11. Was there any particular person or thing that drew your attention the most?

* ask only for stress condition

**Questions List Two**

Confer Group – YES/NO

Number Identifier…………………..

This interview is conducted between _________________________________

and _________________________.The time is now _______________.The

date is _________________.

*Do you agree to take part in this interview?*

*Did you confer with your partner? ______________________________*

***Free Recall Stage***

*Explain the incident from the time that you entered the room.*

***Questions***

CQ9. What were your main thoughts during the incident?

CQ4. How did you feel?

CQ1. Upon entering the room, what did you immediately focus on?

PQ15. Describe any other people present.

PQ14. What was the lighting like in the room?

*PQ12.Where there any shots fired in the incident?

CQ7. Describe your movements in the room.

PQ18. What items were in the room?

CQ6. Describe any persons you observed.

CQ3. Describe any sounds you heard.

PQ11. How far away where you from the persons?

*CQ8. Did you use of force? And if so why was that necessary?

PQ13. What was your partner doing when you were in the room?

PQ19. What did you say to your partner?

PQ20. How long did the event take?

*CQ5. Can you describe any of the weapons you may have seen.

CQ2. What did you observe?

*CQ10.Was your use of force decision successful?

PQ17. Can you describe the appearance of the room?

PQ16. Were you feeling stressed or unable to cope at any time?

* ask only for stress condition

**Supplementary File B**

*In answer to the question, "How did you feel?" in the stress condition of the studies, a random sample of responses is presented:*

- The adrenaline was pumping, it’s hard to say, I didn’t truly feel anything in that situation.It was all over, then you feel a bit shaky, a bit jittery, but when it is happening you just act, you just do whatever your taught.No, I didn’t truly feel anything.

- Relieved to find the offenders.I think your stress levels get higher the further you go into a house when you haven’t found anybody yet.

- Pretty stressed, very elevated heart rate, I felt that prior to going into it. As soon as we were told about multiple offenders with firearms, my heart rate did go up.

- A high level of adrenaline and especially just before going in that adrenaline was at a very high level.In addition, there was some nervousness about I’m going to be in there that this was a life or death situation.We went in there very dynamically to effect an arrest and not get killed.

- Anxious, my adrenaline was up, everything seemed pretty clear although, I saw the firearm quickly, reacted quickly, and then moved straight from him to the next offender without any sort of pause or delay in actions.

- It’s kind of hard, if he is pointing a gun at me, then, I was at a heightened state of arousal in terms of heart rate.I don’t think I was nervous, I was eager, I kind of enjoy, I enjoyed doing it, the fact that we were, I didn’t know what was going on, and it was a training exercise and the unknown was kind of exciting.

- (long pause) I don’t know truly, felt an adrenaline pump, I felt I needed to stop any threats.I didn’t feel any fear, I needed to get the job done type of feeling.

- Confused, I didn’t know the room was, the scenarios, the demeanour of the suspects, the intention of the suspects.

- Quite serious, it almost felt it was quite slow in a way, the male that I shot at was turning toward me in a truly slow and deliberate manner.I felt I was obligated to prevent any further action by him, and when I had the stoppage, my first concern was to look after my partner because she had been left alone, as I had moved away behind a wall.I was more concerned that she was OK than that I had shot the male.Everything kind of narrowed, I was a bit warm, breathing heavily, and it fluctuated between seeing everything in the room and then the males, focusing on my partner, focusing on my firearm, I had tunnel vision, so all I saw was my gun nothing else, and then my partner, nothing else.Whenever I looked at anything, it was always specifically that object, item or person.

It felt a threat to me.

Nervous.

A bit of stress when I saw the two offenders.

I feel at any moment…I felt I must take him down.

A bit nervous.

I thought it was going to be dangerous.

I feel a bit stressed, but I managed to help my partner

*In answer to the question, "Were you feeling stressed or unable to cope at any time?" The following comments were randomly collected from participants in the Stress Condition:*

Yes.

Yes, I just talked it through.

- I felt in control, relatively calm, you are more focused, definitely.

- Look, heightened I suppose, there is always pressure to perform well, we run the scenarios a lot so there is a little bit of inoculation in the scenario environment and it’s a familiar facility, and there’s pressure to perform and I didn’t want to get shot more than I didn’t want to lose.I was satisfied with the outcome.

- I was having a lot of stress yea, I felt capable of performing, at no time did I feel, there was no equipment failure or tactical failure.Just heightened anxiety.

- Not unable to cope, just a heightened state of awareness.

- there was a lot of adrenaline, I was ok.

Yes, especially as he had the gun pointed at me, so I moved back out of the way behind the edge of the door.My partner came out and shot him, I saw him shoot.

That they were a threat to us, I felt a little bit under stress, when I entered, I saw the shotgun and then the pistol.

**Supplementary File C**

Example of completed Study 1 Grading Chart

No 15/Interview/Stress Scenario/2nd Investigation

| Objects in Experimental Room | C/P | Correct | Incorrect |
| --- | --- | --- | --- |
| Weed spray on floor near entrance and inside door |  |  |  |
| Settee |  |  |  |
| Filing Cabinet - dark gray |  |  |  |
| Round Table |  |  |  |
| Rectangular Table |  |  |  |
| Chairs near rec table x 2 |  |  |  |
| Chair near round table x 1 |  |  |  |
| Video camera on small tripod on rectangular table | P | x |  |
| 2 x boxing gloves blue in color on rectangular table |  |  |  |
| Plastic multi coloured model on stand on rectangular table |  |  |  |
| Small dumbbell black on floor near rectangular table |  |  |  |
| Dumbbell with 3 weights on chrome bar on floor near round table |  |  |  |
| Red medicine ball on round table |  |  |  |
| Telephone with receiver lying on round table |  |  |  |
| Chair next to settee |  |  |  |
| Australian flag draped over settee back |  |  |  |
| On filing cabinet - tv remote |  |  |  |
| On filing cabinet - bag mitts |  |  |  |
| On filing cabinet - wooden item with leather covering |  |  |  |
| On filing cabinet - small plastic container |  |  |  |
| On filing cabinet - roll of white tape |  |  |  |
| On round table -tin of blue 'RAID' fly spray |  |  |  |
| Orange reflective vest on settee back next to flag |  |  |  |
| Black canvas bag on settee |  |  |  |
| Flat screen tv on wall in corner (near window) |  |  |  |
| White plastic bin with large red exercise ball on top under tv |  |  |  |
| Video camera on tripod facing into room, located under TV |  |  |  |
| Kitchen leading from main room on right | P | x |  |
| White fridge in kitchen near main room |  |  |  |
| Air Conditioner on back wall above rectangular table |  |  |  |
| AC remote fixed on wall below air conditioner |  |  |  |
| Sports drink bottle, green in color on rectangular table |  |  |  |
|  |  |  |  |
| Environment |  |  |  |
| Well lit, natural light | P | x |  |
| Main window on left |  |  |  |
| White roller blinds on main windows of room | P | x |  |

| Person on Right Description in Experimental Room | C/P | Correct | Incorrect | | |
| --- | --- | --- | --- | --- | --- |
| Black face mask | C |  | |  |  |
| Short sleeve shirt- patterned with white lines in small shapes containing multi coloured center, beiges, light blues, reddish-brown. Buttoned at front with collar. Fully visible worn over waistband. | C |  | |  |  |
| Long sleeve dark green fleece upper garment, lower part of sleeves visible from the short sleeves of the over shirt to the hands |  |  | |  |  |
| Dark blue long trousers | C |  | |  |  |
| Dark gray and black footwear, laced in a small boot design |  |  | |  |  |
| Hands visible |  |  | |  |  |
| White undershirt visible around the neck area |  |  | |  |  |
|  |  |  | |  |  |
| Other Distinguishing Features |  |  | |  |  |
| Height 183 - 185 cm |  |  | |  |  |
| Weight 84 - 90 kg |  |  | |  |  |
| White Caucasian |  |  | |  |  |
| Slim build |  |  | |  |  |
| Weapon Description |  |  | |  |  |
| 9 mm Glock semiauto |  |  | |  |  |
| Blue frame |  |  | |  |  |
| Black slide |  |  | |  |  |
|  |  |  | |  |  |
| Actions - Stress Scenario |  |  | |  |  |
| Offender sitting in chair on right side of room as police enter. | C | x | |  |  |
| Offender rises from chair and stands |  |  | |  |  |
| Offender moves his right hand toward pistol that is on top of round table | C | x | |  |  |
| Offender places right hand on pistol |  |  | |  |  |
| Offender looks toward police |  |  | |  |  |
| Offender moves pistol up off table |  |  | |  |  |
| Offender moves pistol muzzle toward police |  |  | |  |  |
| Offender obeys police commands |  |  | |  |  |
| Offender is shot | C | x | |  |  |
| Offender is not shot |  |  | |  |  |
| Offender goes to ground |  |  | |  |  |
|  |  |  | |  |  |
| Simulated participant on Right - Control Scenario |  |  | |  |  |
|  |  |  | |  |  |
| Person on right sitting in chair near round table |  |  | |  |  |
| Greets visiting police officers |  |  | |  |  |
| Informs police "These envelopes contain a course of fire and scenarios" |  |  | |  |  |
| Informs police "Take them outside and read them" |  |  | |  |  |
| Hands 2 envelopes to nearest police officer |  |  | |  |  |
|  |  |  | |  |  |
| Note: Facial features visible |  |  | |  |  |
| Clean shaven |  |  | |  |  |
| Dark brown hair, stylish cut, collar length |  |  | |  |  |
|  |  |  | |  |  |
|  |  |  | |  |  |
|  |  |  | |  |  |
|  |  |  | |  |  |

| Person Description on left in Experimental Room | C/P | Correct | Incorrect |
| --- | --- | --- | --- |
| Black face mask | C | x |  |
| Short sleeve green and black with small white patches football shirt with yellow and red motif on right side chest area, black written motif on left side chest area | C | x |  |
| Long sleeve dark green fleece upper garment, lower part of sleeves visible from the short sleeves of the over shirt to the hands | C | x |  |
| Dark blue long trousers | C | x |  |
| Black footwear, laced in a small boot design |  |  |  |
| Hands covered by boxing bag mitts |  |  |  |
| White undershirt visible around the neck area |  |  |  |
|  |  |  |  |
| Other Distinguishing Features |  |  |  |
| Height 183 - 185 cm |  |  |  |
| Weight 90 - 95 kg |  |  |  |
| White Caucasian |  |  |  |
| Large Athletic Build |  |  |  |
| Weapon Description |  |  |  |
| Black shotgun (Remington 870) long barrel - (Benelli 18) | C |  | x |
| Yellow tape on barrel (4 inch from muzzle) |  |  |  |
| Pistol grip model | C | x |  |
|  |  |  |  |
| Actions Stress Scenario |  |  |  |
|  |  |  |  |
| Offender facing into left corner of room | C | x |  |
| Offender commences to turn to his right, remaining in the same area | C | x |  |
| Offender raises weapon from a concealed position occluded by his body | C | x |  |
| Offender continues to turn toward police still raising weapon |  |  |  |
| Offender points the muzzle of the weapon in the direction of police |  |  |  |
| Offender only uses right hand on weapon |  |  |  |
| Offender does not speak |  |  |  |
| Offender falls (if engaged) onto his front | C | x |  |
|  |  |  |  |
| Simulated participant on Left - Control Scenario |  |  |  |
|  |  |  |  |
| Simulated participant sitting in chair on left side of room next to rectangular table |  |  |  |
| Simulated participant reading a magazine facing into the room (toward police) |  |  |  |
| Simulated participant does not engage in conversation |  |  |  |
| Note: Facial features visible |  |  |  |
| Shaven head |  |  |  |
| Clean shaven |  |  |  |
| Light coloured features |  |  |  |
|  |  |  |  |
| **Sounds** |  |  |  |
| Participant speech | C | x |  |
| Speech heard from partner | C | x |  |
| Speech heard from simulated participants |  |  |  |
| Sound from object (s) (e.g.,firearms) | C | x |  |
